# Immuno-neural mechanisms in gastrointestinal tumorigenesis: bridging inflammation, neural regulation, and therapeutic innovation

**DOI:** 10.3389/fimmu.2025.1682356

**Published:** 2026-02-20

**Authors:** Yan Zhao, Ji-feng Sui, Dong-ning Wu, Min Chen, Jin-xia Ni, Yue Zhang, Shi-yong Xin, Meng-Nan Fan

**Affiliations:** 1 The Second Affiliated Hospital of Liaoning University of Traditional Chinese Medicine, Shenyang, China; 2 ICU Ward, The Affiliated Hospital of Liaoning University of Traditional Chinese Medicine, Shenyang, China; 3 Faculty of Chinese Medicine and State Key Laboratory of Quality Research in Chinese Medicines, Macau University of Science and Technology, Macao, Macao SAR, China; 4 Dongzhimen Hospital, Beijing University of Chinese Medicine, Beijing, China; 5 Guang’anmen Hospital, China Academy of Chinese Medical Sciences, Beijing, China

**Keywords:** cancer neuroscience, chronic inflammation, gastrointestinal tumors, immune checkpoint inhibitors, neuro-immune crosstalk, tumor microenvironment (TME)

## Abstract

Gastrointestinal (GI) tumors remain a leading cause of global cancer mortality, with late-stage diagnosis and metastatic dissemination posing major clinical challenges. This review synthesizes current understanding of the intricate interplay between immune regulation, neural signaling, and tumor microenvironment dynamics in GI malignancies. We highlight how chronic inflammation, driven by pathogens like *H. pylori* or inflammatory bowel disease, establishes a pro-tumorigenic milieu through cytokine networks (IL-1β, TNF-α, IL-6) and Wnt/β-catenin signaling, while neural components (serotonergic, cholinergic, and peptidergic pathways) actively participate in cancer progression via neurotrophic factors and neurotransmitter-mediated crosstalk. Emerging evidence reveals that colorectal cancer stem cells exploit neuronal signaling (particularly 5-HT/Wnt activation) for self-renewal, and that perineural invasion serves as a critical metastatic route. The dual role of immune cells is explored, with macrophages (M1/M2 polarization), T cells, and neutrophils exhibiting both tumor-suppressive and pro-metastatic functions depending on context. We evaluate recent therapeutic advances including immune checkpoint inhibitors, CAR T-cell therapies, and neural-targeted approaches, while addressing limitations such as chemoresistance and immune-related adverse events. The potential of microbiota modulation and nanotechnology for precision therapy is discussed. By integrating molecular mechanisms with clinical observations, this work proposes that combinatorial strategies targeting immuno-neural axes may overcome current treatment barriers, emphasizing the need for early detection and personalized approaches in GI oncology.

## Introduction

According to GLOBOCAN 2022 data, the global cancer burden continues to escalate, with approximately 20 million new cases and 9.7 million deaths reported in 2022 (1). Gastrointestinal malignancies occupy a prominent position: colorectal cancer ranks third globally with 1.926 million new cases, while gastric cancer ranks fifth with 968,000 cases (1, 2). Combined, these malignancies account for approximately 1.56 million deaths, representing over 16% of total global cancer mortality (1, 2).

Gastrointestinal tumors exhibit striking geographic disparities, with East Asia bearing the heaviest disease burden—China alone accounts for nearly half of all gastric cancer cases worldwide (3). These disparities are closely associated with dietary habits, *Helicobacter pylori* infection rates, and genetic susceptibility (2, 4). Alarmingly, despite an overall decline in age-standardized incidence rates, early-onset gastrointestinal malignancies in individuals under 50 years of age show an increasing trend, potentially linked to modern lifestyle factors and the obesity epidemic (3).

Currently, most patients are still diagnosed at advanced stages. The 5-year survival rate for advanced gastric cancer remains below 10%, whereas early diagnosis can elevate survival rates to over 90% (5). This substantial disparity underscores the urgent need for deeper understanding of tumorigenesis mechanisms, development of early detection biomarkers, and innovative therapeutic strategies.

The gastrointestinal tract represents the body’s largest immune organ, harboring the majority of the body’s immune cells, including macrophages, T lymphocytes, and natural killer cells (6). This unique immunological landscape makes the gastrointestinal tract an ideal model for investigating tumor immunology.

The adaptive immune system plays a critical role in tumor surveillance through T cell-mediated cytotoxicity. CD8^+^ cytotoxic T lymphocytes recognize tumor antigens and directly eliminate cancer cells, while CD4^+^ helper T cells orchestrate the overall anti-tumor response (6). The tumor microenvironment (TME) has emerged as a key determinant of prognosis—high-density tumor-infiltrating lymphocytes are generally associated with favorable outcomes (7). The innate immune system likewise plays a dual role: macrophages can polarize into anti-tumorigenic M1 or pro-tumorigenic M2 phenotypes; infiltration of tumor-associated neutrophils and regulatory T cells typically correlates with immunosuppression and poor prognosis (2, 8).

Advances in immunotherapy have significantly transformed the therapeutic landscape of gastrointestinal malignancies. CAR-T cell therapy and bispecific T-cell engagers harness engineered T cells to specifically recognize tumor antigens (9); immune checkpoint inhibitors have achieved breakthrough progress in microsatellite instability-high colorectal cancer and selected gastric cancers (5). However, most patients exhibit limited responses to immunotherapy, prompting researchers to explore the regulatory role of the nervous system on the immune microenvironment.

Cancer neuroscience, as an emerging interdisciplinary field, is unveiling the crucial role of the nervous system in tumor initiation and progression. A landmark review published in *Cell* in 2023 defined it as the discipline studying bidirectional interactions between the nervous system and tumors (10).

The gastrointestinal tract possesses a unique neural innervation system. The enteric nervous system, termed the “second brain,” contains approximately 500 million neurons capable of regulating gastrointestinal function independently of the central nervous system (11). The sympathetic and vagus nerves form an extrinsic neural network that not only regulates digestive function but also profoundly participates in shaping the tumor microenvironment. Perineural invasion—the infiltration and spread of tumor cells along nerve fibers—has been identified as a significant prognostic factor in various gastrointestinal malignancies (12). Neurotrophic factors such as NGF, BDNF, and GDNF play pivotal mediating roles in tumor-neural interactions (13).

Recent studies have revealed direct pro-tumorigenic effects of neurotransmitters. Research by Zhi and colleagues published in *Nature* demonstrated that nociceptive sensory neurons expressing CGRP promote gastric cancer progression through the CGRP-RAMP1 axis (14). Serotonin reprograms lipid metabolism and suppresses ferroptosis via the HTR2B receptor, correlating with poor prognosis in gastric cancer (15). In colorectal cancer, 5-HT released by enteric serotonergic neurons activates Wnt signaling pathways, promoting cancer stem cell self-renewal (16).

In contrast to pro-tumorigenic effects, vagal nerve signaling can exert protective effects through the cholinergic anti-inflammatory pathway. Studies have shown that vagotomy significantly increases peritoneal metastasis in murine models while altering the composition of tumor-associated immune cells (17). These findings suggest that different neural signals may exert contrasting effects on tumors.

Bidirectional communication between the immune and nervous systems plays a critical role in the tumor microenvironment. The nervous system regulates immune cell proliferation, migration, and effector functions through the release of neurotransmitters and neuropeptides (18). Cytokines produced by immune cells can reciprocally influence neuronal activity, forming bidirectional feedback loops (19).

Sun and colleagues systematically elucidated recent advances in neuro-immune crosstalk in digestive system tumors, emphasizing that tumor cells create a dynamically regulated microenvironment within the neuro-immune network by sharing key signaling molecules such as neurotransmitter receptors and immune checkpoint proteins (18). Integrating cancer neuroscience with tumor immunology holds significant importance for elucidating tumor mechanisms and developing novel therapeutic strategies.

This review aims to provide an integrative framework elucidating the convergence mechanisms of immune and neural regulation in gastrointestinal tumors. We will first introduce the molecular basis of the immune-neural axis; subsequently explore chronic inflammation as an initiating factor of dysregulation; then analyze specific mechanisms of immune-neural interactions in gastric and colorectal cancers; and finally evaluate innovations in targeted therapies and prospects for clinical translation. Through this integrative perspective, we hope to establish a theoretical foundation for developing more effective combination therapeutic strategies.

## 2 Molecular basis of the immune-neural axis

### 2.1 Shared signaling molecules and receptors

Communication between the immune and nervous systems relies on a repertoire of shared signaling molecules and receptors. Nearly all immune cells express at least one neurotransmitter receptor, enabling direct neural modulation of immune function (20).

#### 2.1.1 Serotonin receptor system

Serotonin (5-hydroxytryptamine, 5-HT) is predominantly synthesized in enterochromaffin cells of the gastrointestinal tract, accounting for approximately 95% of total body serotonin. To date, 14 5-HT receptor subtypes have been identified, classified into 7 families (5-HT1-7), with multiple subtypes expressed on immune cells (18, 20).

Monocytes and macrophages express 5-HT2B and 5-HT7 receptors, through which serotonin modulates cytokine secretion and promotes M2 macrophage polarization (18). Dendritic cells primarily express the 5-HT2B receptor; its activation suppresses TLR-induced pro-inflammatory cytokine production (TNF-α, IL-6, IL-8, IL-12) without affecting type I interferon responses (21). T cells express multiple 5-HT receptor subtypes, and serotonergic signaling regulates T cell proliferation, differentiation, and cytokine production (18). In the tumor microenvironment, dysregulated 5-HT signaling may promote tumor immune evasion by modulating immune cell function.

#### 2.1.2 Adrenergic receptor system

Both innate and adaptive immune cells express adrenergic receptors, predominantly the β2-adrenergic receptor (ADRB2), enabling direct responsiveness to sympathetic nervous system signals (19). Norepinephrine (NE) released from sympathetic nerves regulates cell migration, cytokine secretion, and cytotoxic activity through β2 receptor activation on immune cells.

In the tumor microenvironment, catecholamine neurotransmitters promote the formation of an immunosuppressive milieu via β-adrenergic receptor activation, inhibiting cytotoxic T cell and NK cell activity, thereby impairing anti-tumor immune surveillance (19). NE can also activate tumor-associated fibroblasts, enhancing extracellular matrix secretion and promoting tumor progression (22). These findings suggest that chronic stress-induced sympathetic nervous system hyperactivation may facilitate tumor development through immunosuppressive mechanisms.

#### 2.1.3 Cholinergic receptor system

Acetylcholine (ACh) is produced not only by neurons but also synthesized and released by T cells, with CD4^+^ T cells containing higher ACh levels than CD8^+^ T cells (6). Nicotinic acetylcholine receptors (nAChRs) on immune cells, particularly the α7 subtype (α7nAChR), serve as key mediators of neuro-immune communication.

Expression of α7nAChR on macrophages constitutes the molecular foundation of the cholinergic anti-inflammatory pathway (CAP) (23). Activation of this receptor suppresses pro-inflammatory cytokine production (TNF-α, IL-1β, IL-6) and has demonstrated protective effects in various inflammatory disease and cancer models (24). The discovery of this pathway provides a theoretical basis for vagus nerve stimulation as an anti-tumor strategy.

#### 2.1.4 Neuroactive substances produced by immune cells

Immune cells not only express neurotransmitter receptors but also synthesize various neuroactive substances. Mast cells represent an important non-neuronal source of serotonin; their released 5-HT can influence local neural function and immune responses (18). Macrophages and T cells can produce substance P and calcitonin gene-related peptide (CGRP), molecules that possess both neuromodulatory functions and regulate immune cell activity (10). This bidirectional communication capacity establishes immune cells as integral components of the neuro-immune axis.

### 2.2 Convergence of key signaling pathways

Immune and neural regulation share multiple key signaling pathways, and their convergence provides a molecular foundation for understanding neuro-immune interactions in the tumor microenvironment.

#### 2.2.1 Wnt/β-Catenin pathway

The Wnt/β-catenin signaling pathway is a critical regulator of intestinal epithelial renewal and serves as a core driver of colorectal carcinogenesis—approximately 90% of colorectal cancers harbor mutations in this pathway (25). Notably, this pathway represents a crucial intersection point for neural signaling and immune regulation.

In colorectal cancer stem cells (CSCs), 5-HT derived from enteric serotonergic neurons activates 5-HT receptors on CSC surfaces, initiating Wnt signaling to promote stem cell self-renewal and tumorigenesis (16). This discovery directly links neural signaling to cancer stem cell properties. Concurrently, inflammatory signals such as the PI3K/AKT pathway can directly influence β-catenin accumulation and stability, while cytokines (e.g., TNF) amplify β-catenin transduction by activating epithelial ERK and AKT signaling (25, 26). Thus, the Wnt/β-catenin pathway integrates multiple signals from neurotransmitters, growth factors, and inflammatory cytokines.

#### 2.2.2 STAT3 pathway

Signal transducer and activator of transcription 3 (STAT3) serves as a common downstream effector of cytokines and neurotrophic factors. IL-6 family cytokines (IL-6, IL-11, IL-22) and hepatocyte growth factor family members all activate STAT3 (7).

STAT3 plays a pivotal role in colitis-associated carcinogenesis. IL-6 promotes colorectal cancer cell proliferation and metastasis through STAT3 activation, while simultaneously mediating intestinal epithelial cell healing, exemplifying the dual nature of inflammatory signaling (7). Neurotrophic factors such as NGF and BDNF can also activate STAT3 signaling through their respective receptors TrkA and TrkB, suggesting that neural and inflammatory signals may achieve functional integration through STAT3 (13).

#### 2.2.3 NF-κB pathway

Nuclear factor kappa B (NF-κB) serves as the master regulator of inflammatory responses and is also an important modulator of neural plasticity. In immune cells, NF-κB activation promotes expression of pro-inflammatory cytokines, chemokines, and adhesion molecules; in the nervous system, NF-κB participates in neuronal survival and synaptic plasticity regulation (27).

The mechanism of the cholinergic anti-inflammatory pathway involves NF-κB inhibition. α7nAChR activation suppresses NF-κB activity through multiple pathways including JAK2/STAT3, PI3K/AKT, and MAPK/ERK, reducing pro-inflammatory cytokine production (24). This mechanism explains the anti-inflammatory and potential anti-tumor effects of vagal nerve signaling. Additionally, mutant p53 can promote chronic inflammation and inflammation-associated colorectal cancer by prolonging NF-κB activation (27), integrating genetic mutations, inflammatory signaling, and tumorigenesis into a unified molecular framework.

### 2.3 Anatomical basis of neuro-immune communication

Interactions between the nervous and immune systems depend not only on molecular mechanisms but also require anatomical proximity. The unique tissue architecture of the gastrointestinal tract provides an ideal physical substrate for neuro-immune communication.

#### 2.3.1 Spatial relationship between the enteric nervous system and gut-associated lymphoid tissue

Gut-associated lymphoid tissue (GALT) represents the largest immune tissue in the human body, comprising Peyer’s patches, isolated lymphoid follicles, mesenteric lymph nodes, and other structures (28). These structures are distributed along the gastrointestinal tract and form intimate anatomical associations with the enteric nervous system.

Peyer’s patches serve as the primary immune sensing sites in the small intestine, consisting of follicle-associated epithelium (containing M cells), B cell follicles, T cell zones, and dendritic cell-enriched subepithelial dome regions (28). Importantly, Peyer’s patches are innervated by the enteric nervous system, and neuro-lymphoid tissue crosstalk is critical for maintaining intestinal immune homeostasis and regulating responses to commensal bacteria and pathogens (29).

Direct functional connections exist between enteric neurons and immune cells. Enteric neurons can release neuropeptides (such as VIP, NPY, substance P, and CGRP) that act on adjacent immune cells, modulating their activation state and effector functions (10). Conversely, cytokines produced by immune cells can influence enteric neuronal excitability and neurotransmitter release. This bidirectional communication plays a crucial role in inflammatory bowel disease and colitis-associated carcinogenesis (29).

#### 2.3.2 Vagal anti-inflammatory pathway

The vagus nerve is the only parasympathetic pathway connecting the brain to thoracic and abdominal organs, exerting powerful immunomodulatory functions through the cholinergic anti-inflammatory pathway (23). The discovery of this pathway represents a major milestone in the field of neuroimmunology.

The anatomical basis of the cholinergic anti-inflammatory pathway involves complex neural circuitry. Vagal afferent fibers sense peripheral inflammatory signals and transmit them to the nucleus tractus solitarius in the brainstem, which subsequently conveys anti-inflammatory signals back to the periphery through efferent fibers (23). Rather than directly innervating the spleen, the vagus nerve establishes synaptic connections with the sympathetic nervous system via the celiac ganglion. Postganglionic sympathetic nerve fibers release norepinephrine, which acts on β2-adrenergic receptors of choline acetyltransferase-expressing T cells, inducing these T cells to release acetylcholine (24). ACh subsequently acts on α7nAChR on splenic macrophages, suppressing production of pro-inflammatory cytokines such as TNF-α.

In the cancer context, reduced vagal nerve activity is associated with poor prognosis across multiple malignancies. Heart rate variability (HRV), serving as an indicator of vagal activity, is decreased in advanced cancer patients and correlates with survival (30). Animal experiments demonstrate that vagotomy exacerbates gastric and colorectal cancer progression, accompanied by altered tumor-associated macrophage phenotypes and increased pro-inflammatory cytokines (17, 30). These findings suggest that preserving or enhancing vagal function may represent a potential anti-tumor strategy.

Vagus nerve stimulation (VNS), as a neuromodulation technique, has been proposed for cancer therapy. VNS can reduce levels of pro-inflammatory cytokines including IL-6, TNF-α, and IL-1β through α7nAChR activation, potentially converting immunologically resistant (“cold”) tumors into immunologically responsive (“hot”) tumors (31). Non-invasive transcutaneous VNS may emerge as an effective adjuvant treatment option for elderly or frail cancer patients (32).

## 3 Chronic inflammation: the starting point of immune-neural dysregulation

### 3.1 Pathogen-driven inflammation and tumorigenesis

The association between chronic inflammation and carcinogenesis is particularly prominent in the gastrointestinal tract, where pathogenic microbial infection serves as a major trigger for establishing persistent inflammatory states.

#### 3.1.1 *Helicobacter pylori* infection and gastric cancer

*Helicobacter pylori* represents the primary risk factor for gastric cancer development, with infected individuals exhibiting approximately 3-fold increased gastric cancer risk (3). Following colonization of the gastric mucosa, this bacterium induces a series of pathological changes, progressing from chronic gastritis through atrophy and intestinal metaplasia, ultimately leading to malignant transformation.

**Figure 1:**
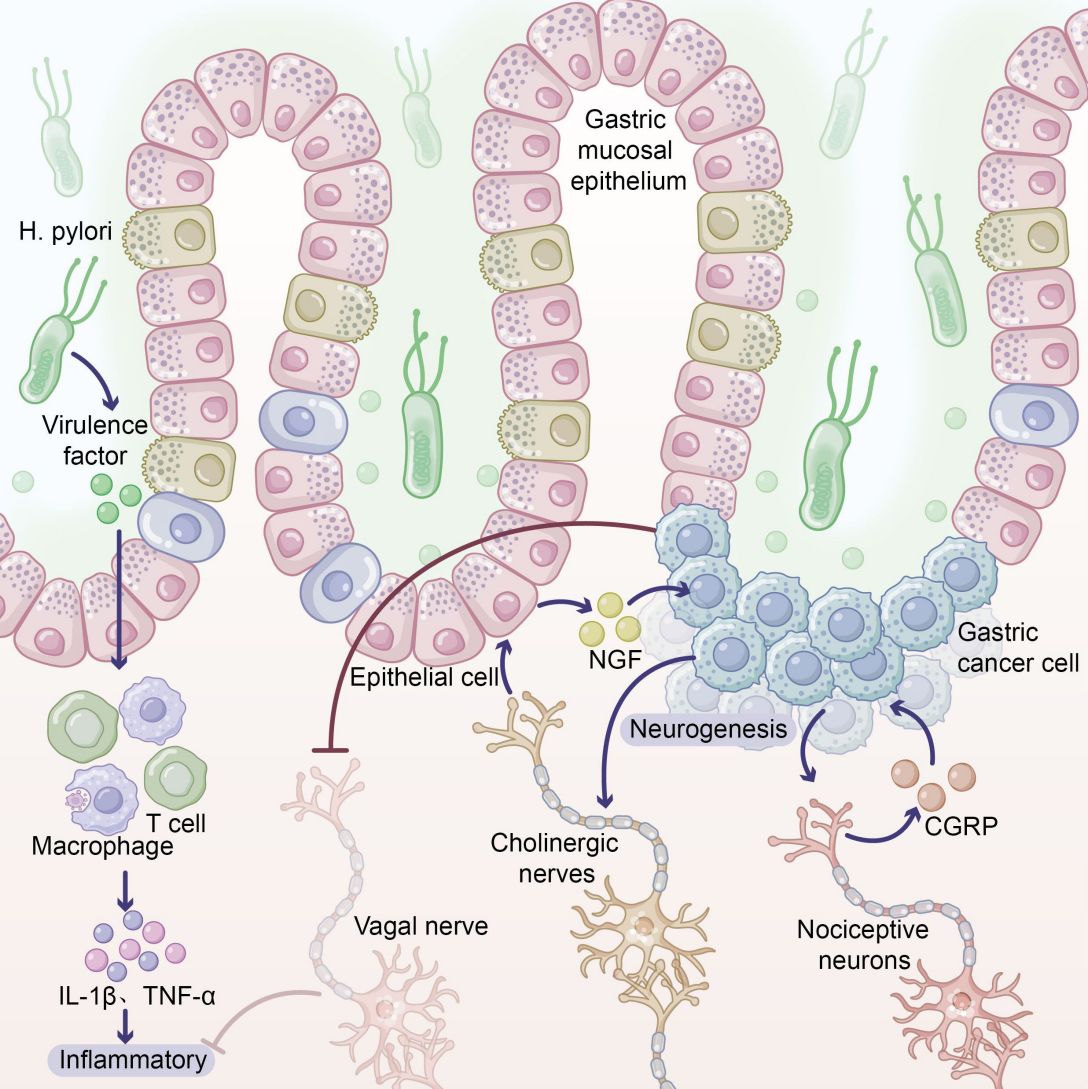
Neuro–immune cascade in Helicobacter pylori–driven gastric carcinogenesis. Schematic representation of how chronic H. pylori infection reshapes the gastric mucosal microenvironment to promote tumor development. Left: H. pylori colonizes the gastric mucosal epithelium and releases virulence factors, activating resident and recruited immune cells, including macrophages and T lymphocytes. These cells secrete pro-inflammatory cytokines such as IL-1β and TNF-α, establishing a chronic inflammatory milieu that favors persistent bacterial survival and progressive epithelial injury. Middle: Inflammatory mediators modulate local neural circuits. Cholinergic nerve fibers stimulate gastric epithelial and emerging gastric cancer cells to overexpress nerve growth factor (NGF), which in turn drives neurogenesis and further ingrowth of cholinergic fibers, creating a pro-tumorigenic feed-forward loop. In parallel, nociceptive neurons release calcitonin gene-related peptide (CGRP), which acts on gastric cancer cells to support their survival and proliferation. Right: The combined effects of chronic inflammation and neural remodeling promote expansion and invasion of gastric cancer cells. A dashed vagal nerve indicates a potentially protective, anti-inflammatory pathway that becomes suppressed or dysregulated within the tumor microenvironment.

As shown in **Figure 1**, the inflammatory microenvironment established by *H. pylori* infection is characterized by elevated IL-1β levels. IL-1β suppresses gastric acid secretion, creating an environment favorable for sustained bacterial proliferation and forming a self-perpetuating vicious cycle (5). Synergistic interactions between IL-1β gene polymorphisms and other cytokine genes (TNF-α, IL-10) can create a high-risk carcinogenic profile, significantly elevating the probability of malignant transformation (3).

T lymphocytes play critical regulatory roles in post-infection gastritis progression. Studies demonstrate that different host immune response types determine infection outcomes: Th1-dominant immune responses lead to more severe gastric mucosal damage, whereas Th2 immune polarization exhibits relative mucosal protective effects (6).

From an integrated neuro-immune perspective, *H. pylori* infection affects not only immune cells but also alters local neural innervation. Research published by Hayakawa and colleagues in *Cancer Cell* demonstrated that cholinergic stimulation induces NGF overexpression in gastric epithelium, promoting neural remodeling with pro-tumorigenic effects (33). Inflammatory mediators such as TNF-α and IL-1β can directly influence neuronal function and survival. Consequently, *H. pylori* infection establishes tissue conditions favorable for tumorigenesis through concurrent remodeling of both immune and neural microenvironments.

#### 3.1.2 Inflammatory bowel disease and colorectal cancer

Inflammatory bowel disease (IBD), encompassing Crohn’s disease and ulcerative colitis, represents a significant risk factor for colorectal cancer development. Patients with long-standing IBD exhibit markedly increased colorectal cancer incidence (8). Malignant transformation risk depends on disease severity, duration, and response to anti-inflammatory therapy.

Animal studies provide direct evidence for inflammation-to-cancer transition. Chemically induced chronic colitis combined with carcinogen exposure leads to multifocal colonic tumors, whereas carcinogen administration following inflammation suppression significantly delays tumorigenesis (8). This indicates that cancer development requires the coexistence of carcinogenic stimuli and a permissive inflammatory microenvironment.

Neuro-immune dysregulation in IBD constitutes an important component of disease pathogenesis. Chronic intestinal inflammation can lead to enteric nervous system damage and dysfunction, including neuronal loss, neurotransmitter imbalance, and altered neural plasticity (11). Conversely, neural dysfunction may compromise intestinal barrier function and immunoregulatory capacity, further exacerbating inflammation. Reduced vagal nerve activity correlates with increased IBD disease activity, suggesting that cholinergic anti-inflammatory pathway insufficiency may participate in disease progression (34). This bidirectional neuro-immune dysregulation creates conditions for inflammation-to-cancer transition.

### 3.2 Neuro-immune crosstalk in the inflammatory microenvironment

**Figure 2:**
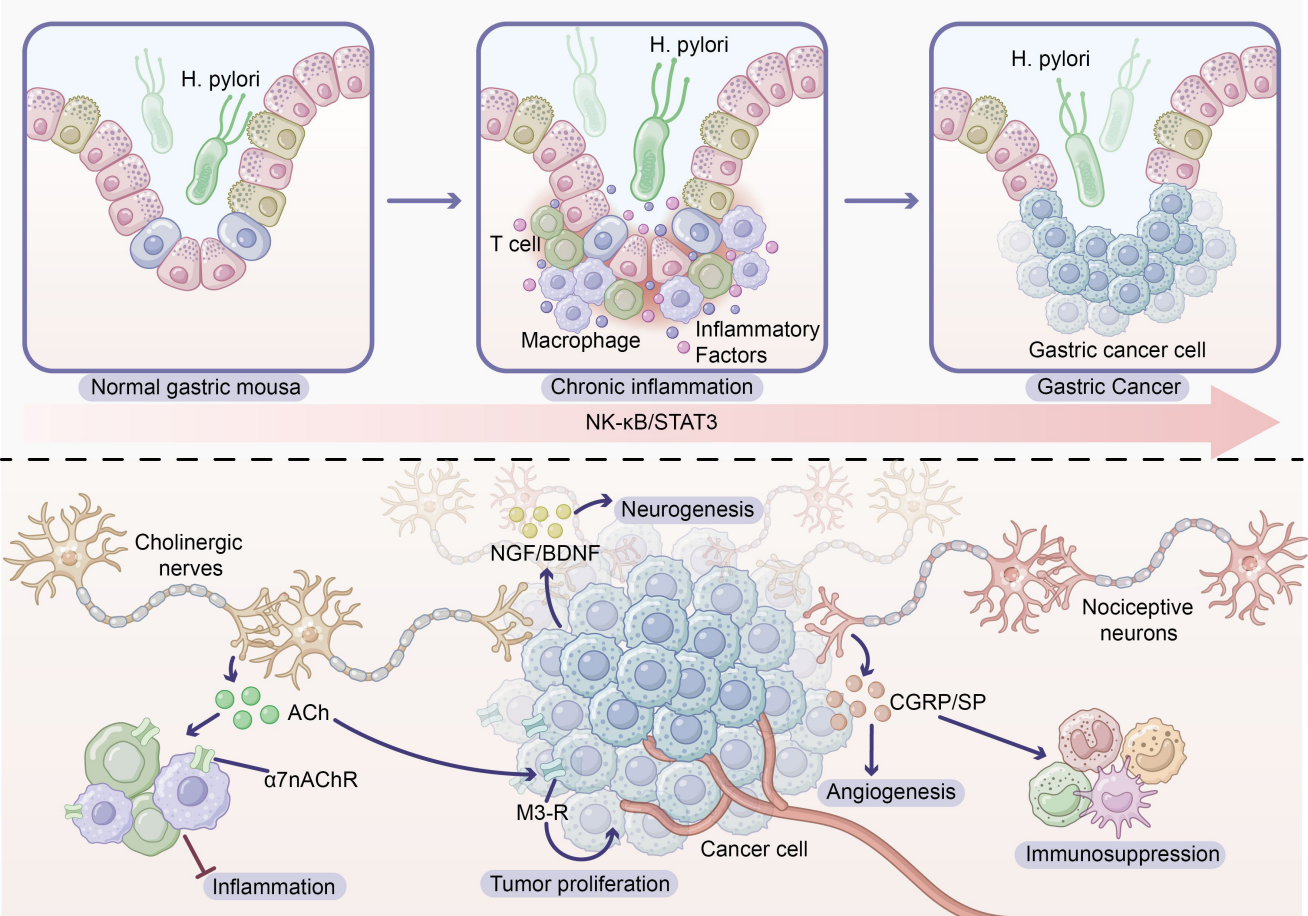
Neural remodeling and inflammation in gastric cancer. Above: Inflammation–cancer sequence in *Helicobacter pylori*–infected stomach. In normal gastric mucosa, chronic infection with *H. pylori* recruits macrophages and T cells and induces a broad “cloud” of inflammatory mediators, leading to persistent gastritis. Sustained activation of NF-κB/STAT3 signaling in this chronic inflammatory milieu drives epithelial transformation and ultimately the emergence of malignant gastric cancer cells. Below: Neural infiltration and neuro–immune crosstalk within the gastric tumor microenvironment. A tumor cell mass is densely innervated by autonomic and sensory fibers, including a prominent nerve branch showing perineural invasion. Tumor and immune cells release neurotrophic factors such as NGF and BDNF, which promote neurogenesis and increased nerve density. Vagal cholinergic fibers release acetylcholine (ACh) that exerts a dual effect: via α7 nicotinic ACh receptors (α7nAChR) on macrophages it suppresses inflammatory responses, whereas via muscarinic M3 receptors on cancer cells it enhances tumor proliferation. Nociceptive neurons secrete neuropeptides, including CGRP and substance P, which support angiogenesis, modulate immune-cell recruitment and polarization, and contribute to local immunosuppression. Together, chronic inflammation–driven neural remodeling and bidirectional neural–immune signaling shape a pro-tumorigenic niche in gastric cancer.

Under conditions of chronic inflammation, complex interaction networks form among immune cells, neural components, and epithelial cells, collectively shaping the pro-tumorigenic microenvironment. We have provided a detailed description of this process in **Figure 2**.

#### 3.2.1 Inflammatory cytokine regulation of neural signaling

Pro-inflammatory cytokines serve not only as mediators of immune responses but can also directly influence neural function. TNF-α, IL-1β, and IL-6 modulate neuronal excitability, neurotransmitter synthesis, and release (18). In gastrointestinal tumors, persistent elevation of these cytokines influences neural signaling through the following mechanisms:

First, inflammatory cytokines upregulate neurotrophic factor expression. TNF-α and IL-1β stimulation induces NGF, BDNF, and GDNF expression in epithelial and immune cells (13). These neurotrophic factors not only promote neural survival and growth but can also directly act on tumor cells expressing corresponding receptors, promoting their proliferation and metastasis.

Second, inflammation alters neurotransmitter receptor expression profiles. Under chronic inflammatory conditions, expression patterns of 5-HT receptors, adrenergic receptors, and cholinergic receptors on immune and epithelial cells may change, affecting cellular sensitivity to neural signals (20).

Third, inflammation-mediated metabolic alterations influence neurotransmitter synthesis. Inflammation activates indoleamine 2,3-dioxygenase (IDO), diverting tryptophan from the 5-HT synthesis pathway to the kynurenine pathway, affecting local 5-HT levels (20).

#### 3.2.2 Neuropeptide effects on immune cell recruitment and polarization

The nervous system actively participates in inflammation regulation and immune cell reprogramming through neuropeptide release. Substance P, CGRP, vasoactive intestinal peptide (VIP), and neuropeptide Y (NPY) play important roles in neuro-immune communication (10).

Substance P, one of the primary neuropeptides released from sensory neurons, promotes mast cell degranulation, enhances macrophage phagocytic activity, and regulates T cell proliferation and cytokine production (10). Under inflammatory and tumor conditions, substance P signaling activation may promote immune cell recruitment and pro-inflammatory phenotypes.

CGRP, the characteristic neuropeptide of nociceptive sensory neurons, has emerged as important in gastric cancer. Research published by Zhi and colleagues in *Nature* demonstrated that peptidergic nociceptive sensory neurons promote gastric cancer progression through the CGRP-RAMP1 axis, with pathway inhibition attenuating tumor growth (14). CGRP also possesses immunomodulatory functions, suppressing DC maturation and T cell proliferation, potentially participating in tumor immune evasion (10).

VIP primarily exerts anti-inflammatory effects, suppressing pro-inflammatory cytokine production by macrophages and DCs while promoting regulatory T cell differentiation (10). In certain tumor types, VIP signaling activation may facilitate establishment of an immunosuppressive microenvironment.

#### 3.2.3 How inflammation simultaneously remodels neural networks and the immune microenvironment

Chronic inflammation simultaneously affects the nervous and immune systems through parallel but interconnected mechanisms, establishing a tissue environment conducive to tumorigenesis.

At the tissue level, persistent inflammation-driven damage and repair cycles lead to tissue remodeling, including fibrosis, angiogenesis, and neural remodeling (35). Tumor-associated macrophages and neutrophils generate reactive oxygen and nitrogen species, exacerbating genomic instability (7). Concurrently, the inflammatory microenvironment promotes neural ingrowth toward tumor regions (tumor innervation), creating conditions for subsequent perineural invasion (12).

At the cellular level, inflammatory signals reprogram immune cell phenotypes. Macrophages in the inflammatory microenvironment tend to polarize toward the M2 phenotype, possessing pro-tumorigenic, pro-angiogenic, and immunosuppressive properties (19). T cells may exhibit functional exhaustion or differentiate toward Tregs, attenuating anti-tumor immunity (22). Simultaneously, neurons and glial cells undergo phenotypic changes under inflammatory stimulation, secreting increased neurotrophic factors and neuropeptides.

At the molecular level, inflammation-activated signaling pathways (such as NF-κB, STAT3, Wnt/β-catenin) integrate multiple inputs from cytokines, neurotransmitters, and growth factors (25, 26). Mutant p53 can promote chronic inflammation and inflammation-associated colorectal cancer by prolonging NF-κB activation (27), integrating genetic mutations, inflammatory signaling, and tumorigenesis into a unified molecular framework.

### 3.3 Clinical value of inflammation-associated biomarkers

Given the central role of inflammation in tumor initiation and progression, inflammation-based biomarkers have become important tools for prognostic assessment in gastrointestinal malignancies.

#### 3.3.1 Systemic inflammation indicators

The neutrophil-to-lymphocyte ratio (NLR) represents one of the most extensively studied inflammatory prognostic indicators. Elevated NLR reflects systemic inflammatory status and immune dysfunction, correlating with poor prognosis across various gastrointestinal malignancies (36). Its prognostic value derives from the dual mechanisms of neutrophil pro-tumorigenic effects (releasing pro-angiogenic factors and matrix metalloproteinases) and attenuated lymphocyte-mediated anti-tumor immunity.

The Glasgow Prognostic Score (GPS), based on C-reactive protein and albumin levels, reflects systemic inflammation and nutritional status and has been validated as an independent prognostic factor in multiple gastrointestinal malignancies including gastric, colorectal, and pancreatic cancers (36). The platelet-to-lymphocyte ratio (PLR) reflects platelet-mediated inflammation and immune function balance; as the primary storage reservoir of serotonin, platelets can release 5-HT at inflammatory and tumor sites, influencing the local microenvironment (20).

#### 3.3.2 Emerging applications of neuro-immune-related markers

With deepening understanding of the neuro-immune axis role in tumors, novel biomarkers are being explored. Heart rate variability (HRV), as a non-invasive indicator of vagal nerve activity, correlates with cancer patient prognosis and may reflect cholinergic anti-inflammatory pathway functional status (30). Low HRV associates with increased systemic inflammation and poor prognosis.

Serum neurotrophic factor levels may serve as potential biomarkers. Studies show that NGF and BDNF are elevated in serum of certain tumor patients, possibly reflecting the degree of tumor-neural interaction activity (13).

Neural density and perineural invasion (PNI) status in tumor tissue have been confirmed as important pathological prognostic factors. High neural density and PNI positivity typically associate with more aggressive tumor behavior and worse prognosis (12). Immunohistochemistry-based detection of neural markers (such as neural cell adhesion molecule NCAM, neurofilament proteins, synaptophysin) may facilitate patient stratification and treatment decision-making.

In summary, chronic inflammation, as the starting point of immune-neural dysregulation, establishes the pro-tumorigenic microenvironment through complex molecular and cellular mechanisms. Understanding these mechanisms not only helps elucidate gastrointestinal tumor pathogenesis but also provides a theoretical foundation for developing prevention and therapeutic strategies targeting the immune-neural axis.

## 4 Immune-neural interactions in gastric cancer

### 4.1 Immune regulatory mechanisms

#### 4.1.1 Role of T lymphocytes in post-*Helicobacter pylori* infection gastritis

*Helicobacter pylori* infection represents a major risk factor for gastric cancer development, with the chronic inflammatory response it mediates playing a critical role in the carcinogenic process. Following *H. pylori* infection, significant infiltration of CD4^+^ T cells occurs in the gastric mucosa, predominantly characterized by activation of Th1 and Th17 cells (37). These T cell subsets promote persistent chronic inflammation through secretion of IFN-γ and IL-17, driving the development of gastric mucosal atrophy and intestinal metaplasia.

Regulatory T cells (Tregs) exert a dual role in *H. pylori*-associated gastritis: on one hand, they provide protection by suppressing excessive inflammatory responses; on the other hand, excessive Treg accumulation may promote immune evasion, creating conditions for malignant transformation (38). Application of single-cell sequencing technology has revealed unique exhausted CD8^+^ T cell subsets in the tumor microenvironment of *H. pylori*-positive gastric cancer patients, with these cells expressing high levels of inhibitory receptors including PD-1, TIM-3, and LAG-3 (5).

#### 4.1.2 Cytokine networks and carcinogenic risk

As shown in **Figure 1**, IL-1β, TNF-α, and IL-6 constitute the core cytokine network driving transformation from *H. pylori*-associated gastritis to gastric cancer. IL-1β, produced by activated macrophages and epithelial cells, promotes gastric acid secretion inhibition and gastric mucosal atrophy through NF-κB signaling pathway activation (7). IL-1β gene polymorphisms significantly correlate with gastric cancer risk, with individuals carrying specific alleles exhibiting 2–3 fold increased gastric cancer risk.

IL-6, as a key molecule linking inflammation to cancer, promotes gastric cancer cell proliferation, invasion, and angiogenesis through JAK2/STAT3 signaling pathway activation (6). Studies demonstrate that serum IL-6 levels significantly correlate with TNM stage, lymph node metastasis, and prognosis in gastric cancer patients. TNF-α plays a critical role in early inflammatory responses to *H. pylori* infection, not only directly damaging gastric epithelial cells but also promoting carcinogenesis-associated gene expression through STAT3 and NF-κB pathway activation (6).

### 4.2 Neural regulatory mechanisms

#### 4.2.1 Neurotrophic factor signaling

Nerve growth factor (NGF) and its receptor system play important roles in gastric cancer development and progression. Research published by Hayakawa and colleagues in *Cancer Cell* demonstrated that NGF and its high-affinity receptor TrkA are significantly upregulated in gastric cancer tissue, with the NGF/TrkA signaling pathway promoting tumor cell survival, proliferation, and invasion through PI3K/Akt and MAPK/ERK pathway activation (33). Importantly, NGF acts not only on neural cells but also directly on tumor cells and immune cells, forming a complex regulatory network.

Brain-derived neurotrophic factor (BDNF) is also significantly upregulated in gastric cancer, promoting tumor growth and metastasis through TrkB receptor activation. Studies have found that BDNF can induce epithelial-mesenchymal transition (EMT) in gastric cancer cells, enhancing their invasive and metastatic capabilities (13). Additionally, neuropeptides such as substance P and calcitonin gene-related peptide (CGRP) are highly expressed in the gastric cancer microenvironment, regulating tumor angiogenesis and immune cell function through binding to their respective receptors (10).

#### 4.2.2 Serotonin/HTR2B pathway and ferroptosis inhibition

Serotonin (5-HT) and its receptor system play important roles in gastric cancer pathophysiology. The gastrointestinal tract represents the body’s largest 5-HT production site, with approximately 95% of 5-HT produced by enterochromaffin cells. Research published by Tu and colleagues in *Cancer Research* found that 5-HT2B receptor (HTR2B) expression is significantly upregulated in gastric cancer tissue, with HTR2B signaling pathway activation promoting tumor cell survival through ferroptosis inhibition (15).

HTR2B-mediated ferroptosis inhibition mechanisms involve several key pathways: HTR2B activation upregulates SLC7A11, a key component of system Xc^-^, enhancing cystine uptake and glutathione synthesis, thereby inhibiting lipid peroxidation (39); simultaneously, it upregulates GPX4 expression through PI3K/Akt pathway activation, directly inhibiting ferroptosis occurrence. This discovery provides new theoretical basis for targeting serotonin signaling pathways in gastric cancer therapy.

#### 4.2.3 Dual role of the vagus nerve

The vagus nerve, as the primary parasympathetic innervation of the stomach, plays important roles in gastric physiological function and pathological processes. Research published by Futoh and colleagues in *Scientific Reports* demonstrated that vagotomy significantly increases peritoneal metastasis in mouse models, suggesting the vagus nerve may exert protective effects (17).

During the acute phase of *H. pylori* infection, vagal nerve activation suppresses pro-inflammatory cytokine release through the cholinergic anti-inflammatory pathway (CAP) (18). Acetylcholine released from the vagus nerve inhibits pro-inflammatory phenotype transformation in macrophages through α7 nicotinic acetylcholine receptor (α7nAChR), reducing tissue damage (24). However, during chronic inflammation and carcinogenesis stages, sustained vagal nerve activation may support tumor growth through promoting M2 macrophage polarization and angiogenesis, demonstrating the stage-dependent nature of vagal nerve effects (19).

### 4.3 Integrated effects of the immune-neural axis

#### 4.3.1 Cholinergic signal regulation of tumor-associated macrophage polarization

Tumor-associated macrophages (TAMs) represent the most abundant immune cell type in the gastric cancer microenvironment, with their polarization state directly influencing tumor progression and therapeutic response. Research has revealed the critical role of neurotransmitters, particularly acetylcholine, in TAM polarization (40). Cholinergic nerve fiber density in gastric cancer tissue positively correlates with M2-type TAM infiltration degree, suggesting neural signals directly participate in immune microenvironment shaping.

Mechanistic studies demonstrate that acetylcholine promotes M2 phenotype polarization through activating α7nAChR on TAM surfaces. α7nAChR signal activation suppresses the NF-κB pathway, reducing pro-inflammatory factor IL-12 and TNF-α secretion while upregulating anti-inflammatory factor IL-10 and TGF-β expression (24). Functionally, neurogenic M2-type TAMs secrete high levels of pro-angiogenic and pro-invasive factors such as VEGF and MMP9, supporting tumor growth and metastasis.

#### 4.3.2 Direct effects of neurotransmitters on T cell function

Accumulating evidence indicates that neurotransmitters can directly regulate T cell functional states. Norepinephrine (NE) in the gastric cancer microenvironment inhibits CD8^+^ T cell cytotoxic function by acting on β2-adrenergic receptors (β2-AR) on T cell surfaces (22). β2-AR signal activation leads to elevated intracellular cAMP levels, inhibiting T cell receptor signal transduction and cytokine production through PKA pathways while upregulating inhibitory receptor expression such as PD-1 (41).

Serotonin’s effects on T cell function exhibit concentration dependence and receptor subtype dependence. Low-concentration 5-HT promotes Th1 differentiation and IFN-γ production through 5-HT1A receptors, whereas high-concentration 5-HT inhibits T cell proliferation and cytokine secretion through 5-HT2A receptors (20). Elevated 5-HT levels in gastric cancer tissue may suppress anti-tumor immune responses through the latter mechanism.

#### 4.3.3 Immune cell-nerve fiber interactions in perineural invasion

Perineural invasion (PNI) represents an important pathological feature of gastric cancer, closely associated with poor prognosis. PNI represents not merely physical tumor cell spread along nerve bundles but rather a dynamic process involving complex interactions among tumor cells, neural cells, and immune cells (12). In PNI regions, dense infiltration of TAMs and neutrophils forms a unique immune microenvironment.

Chemokines released from nerve fibers (such as CX3CL1) recruit monocytes/macrophages to perineural regions through binding to CX3CR1 on immune cell surfaces (42). These immune cells further secrete matrix metalloproteinases such as MMP2 and MMP9, degrading extracellular matrix and creating conditions for tumor cell invasion along nerve bundles. Simultaneously, neurotrophic factors such as NGF and BDNF secreted by TAMs promote nerve fiber sprouting and growth, forming a positive feedback loop of “neural neogenesis-tumor invasion” (43).

T cell function in PNI regions is severely suppressed. CGRP and VIP released from nerve fibers possess potent immunosuppressive effects, inhibiting T cell proliferation, activation, and cytotoxic function through their respective receptors (10).

#### 4.3.4 Tripartite dialogue among nerves, immunity, and tumors in the gastric cancer microenvironment

Integrating the above research findings, a conceptual model can be constructed depicting tripartite dialogue among neural, immune, and tumor cells in the gastric cancer microenvironment. In this model, tumor cells promote neural neogenesis and nerve fiber infiltration through secreting neurotrophic factors such as NGF and BDNF (13, 33). Newly formed nerve fibers release neurotransmitters and neuropeptides that, on one hand, directly act on tumor cells to promote their proliferation and invasion, and on the other hand, regulate immune cell functional states (44).

Specifically, norepinephrine released from sympathetic nerves promotes tumor angiogenesis through β-AR signaling while suppressing anti-tumor functions of CD8^+^ T cells and NK cells (45). Acetylcholine released from parasympathetic nerves promotes M2-type TAM polarization through α7nAChR and may directly stimulate tumor cell proliferation through M3 receptors (33). Research published by Zhi and colleagues in *Nature* demonstrated that CGRP released from sensory nerves promotes gastric cancer progression through RAMP1 receptors while regulating mast cell degranulation and neutrophil recruitment (14).

Immune cells, particularly TAMs, serve as “intermediaries” in the tripartite dialogue. M2-type TAMs not only secrete pro-tumor factors supporting tumor growth but also secrete neurotrophic factors maintaining nerve fiber survival and function (46). This imbalance in neural-immune-tumor tripartite dialogue ultimately promotes gastric cancer progression and metastasis.

## 5 Immune-neural interactions in colorectal cancer

### 5.1 Immune regulatory mechanisms

#### 5.1.1 Immune basis of IBD-associated colorectal cancer

Inflammatory bowel disease (IBD) represents a significant risk factor for colorectal cancer, with ulcerative colitis patients exhibiting 2–5 fold higher colorectal cancer risk compared to the general population (8). IBD-associated colorectal cancer development follows a sequential “inflammation-dysplasia-carcinoma” progression pattern, exhibiting notable differences from sporadic colorectal cancer (see **Figure 2**).

In the chronic inflammatory microenvironment of IBD, excessive activation of CD4^+^ T cells, particularly Th17 cells, represents a hallmark feature. IL-17A secreted by Th17 cells promotes epithelial cell proliferation and anti-apoptotic gene expression through NF-κB and STAT3 signaling pathway activation (47). Innate immune cells, particularly macrophages and neutrophils, play critical roles in IBD-associated carcinogenesis. M1-type macrophages accumulating in inflamed tissue generate substantial reactive oxygen species (ROS), leading to DNA damage and genomic instability (6).

#### 5.1.2 Crosstalk between Wnt/β-Catenin and inflammatory pathways

The Wnt/β-catenin signaling pathway plays a central role in colorectal carcinogenesis, with approximately 80% of colorectal cancers exhibiting pathway aberrant activation due to APC or β-catenin gene mutations (25). In IBD-associated colorectal cancer, chronic inflammation influences Wnt signaling pathway activity through multiple mechanisms.

Pro-inflammatory cytokines such as TNF-α and IL-1β can indirectly promote Wnt signal activation through NF-κB pathway activation. NF-κB and β-catenin possess synergistic binding sites in the promoter regions of multiple target genes, jointly driving expression of pro-proliferative genes including Cyclin D1 and c-Myc (27). Inflammation also influences Wnt signaling pathways through epigenetic modifications; chronic inflammation-induced aberrant DNA methylation can lead to promoter hypermethylation and expression silencing of Wnt antagonists such as DKK1 and SFRP (25).

#### 5.1.3 Pro-tumorigenic effects of key cytokines

IL-6 plays a central role in colorectal cancer development and progression, promoting tumor cell proliferation, anti-apoptosis, and angiogenesis through JAK2/STAT3 signaling pathway activation (7). Epidemiological studies demonstrate that elevated serum IL-6 levels significantly correlate with increased colorectal cancer risk and poor prognosis.

The IL-23/IL-17 axis plays an important role in the colorectal cancer immune microenvironment. IL-23, primarily produced by activated macrophages and dendritic cells, promotes secretion of cytokines including IL-17 and IL-22 through acting on Th17 cells and group 3 innate lymphoid cells (ILC3) (48). IL-17 not only directly stimulates tumor cell proliferation but also recruits MDSCs and neutrophils by inducing chemokine expression, forming an immunosuppressive microenvironment (47).

### 5.2 Neural regulatory mechanisms

#### 5.2.1 Enteric neurons and cancer stem cells: the 5-HT/Wnt axis

The enteric nervous system (ENS) contains approximately 500 million neurons, constituting the “gut brain” system. Research published by Zhu and colleagues in *Neuron* discovered that the ENS plays an important role in colorectal cancer development and progression, particularly in cancer stem cell (CSC) regulation (16).

Enteric neurons represent an important source of intestinal 5-HT. Studies demonstrate that enteric neuron density increases in colorectal cancer tissue, with 5-HT production and release significantly upregulated. 5-HT promotes Wnt/β-catenin signaling pathway activation through activating HTR2B on tumor cell surfaces (16). Mechanistically, HTR2B activation inhibits GSK-3β activity through the Gαq/PKC pathway, preventing β-catenin phosphorylation and degradation, promoting its nuclear translocation and target gene transcription.

This 5-HT/Wnt axis is particularly important in CSC self-renewal and maintenance. LGR5^+^ cells, CSC marker-expressing cells, highly express HTR2B, with 5-HT stimulation significantly enhancing LGR5^+^ cell self-renewal capacity in organoid cultures (16). Pharmacological blockade or genetic knockout of HTR2B can inhibit CSC function and tumor-initiating capacity. These findings suggest that targeting the 5-HT/Wnt axis may represent a novel strategy for CSC elimination.

#### 5.2.2 Differential effects of sympathetic/parasympathetic nerves

Autonomic nervous innervation of the colorectum exhibits complex differential effects in tumor development and progression. Sympathetic nerves primarily exert effects through norepinephrine release, with their role in colorectal cancer remaining controversial. Some studies demonstrate that chronic stress-induced sympathetic nervous system hyperactivation can promote tumor angiogenesis and metastasis through β-AR signaling (22).

However, research published by Balood and colleagues in *Nature* revealed opposite phenomena. Utilizing advanced tissue optical clearing and three-dimensional imaging techniques, they discovered that sympathetic nerve fiber density positively correlates with colorectal cancer patient prognosis, with chemical sympathetic denervation actually accelerating tumor growth (49). This contradiction may arise from dose-dependent and time-dependent sympathetic nervous system effects.

Parasympathetic nerves, primarily the vagus nerve, can directly promote colorectal cancer cell proliferation through acetylcholine release activating M3 muscarinic receptors (50). Additionally, cholinergic signaling suppresses inflammatory responses through α7nAChR; during chronic inflammation stages, this may exert protective effects, but in the tumor microenvironment, it may promote immunosuppression (24).

#### 5.2.3 Clinical significance of perineural invasion

Perineural invasion (PNI) represents an important pathological feature of colorectal cancer, with incidence rates reaching 20-35% in rectal cancer (12). PNI presence significantly correlates with poor tumor differentiation, lymph node metastasis, local recurrence, and poor prognosis.

Molecular mechanism studies have revealed the complex processes underlying PNI occurrence. Tumor cells highly express adhesion molecules such as NCAM and L1CAM, promoting physical contact with nerve fibers (12). Simultaneously, tumor cell-secreted CXCL12 guides tumor cell migration toward nerve bundles through acting on CXCR4 receptors on neural cell surfaces (42).NGF and GDNF released from nerve fibers provide support for tumor cell growth and invasion (15).

### 5.3 Integrated effects of the immune-neural axis

#### 5.3.1 Immune regulation in the enteric neuron-serotonin-Wnt-CSC circuit

**Figure 3:**
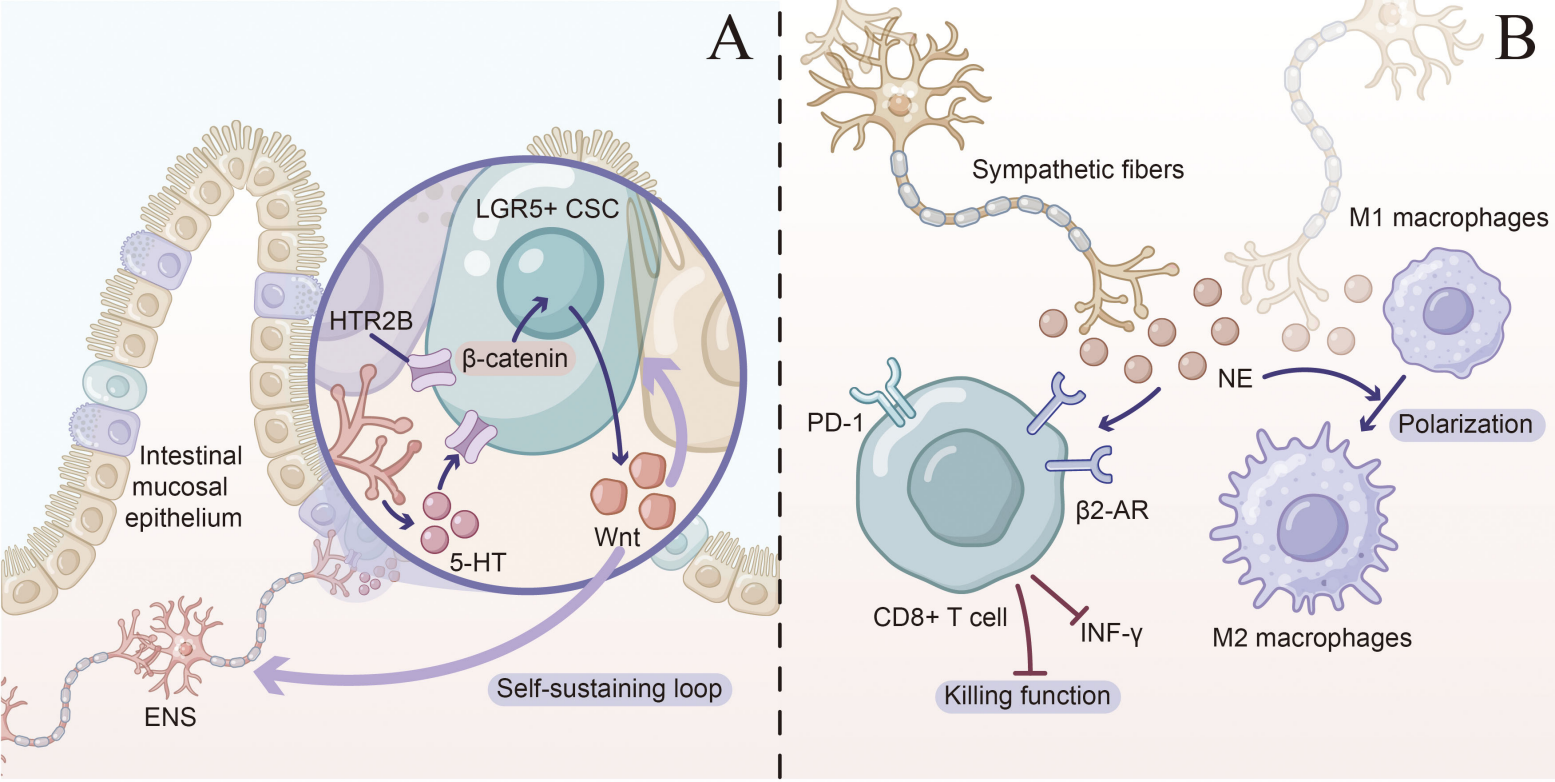
5-HT/Wnt circuitry and neuro–immune regulation in colorectal cancer. **(A)** Enteric neuron–serotonin–Wnt–cancer stem cell self-sustaining loop. In the colorectal mucosa, enteric nervous system (ENS) neurons innervating the intestinal crypt release serotonin (5-HT). Elevated 5-HT acts on LGR5^+^ colorectal cancer stem cells (CSCs) via the 5-HT receptor HTR2B, leading to activation and nuclear translocation of β-catenin and downstream Wnt target gene transcription. Activated CSCs secrete Wnt ligands that further reinforce Wnt/β-catenin signaling within CSCs and/or feedback to ENS components, establishing a self-sustaining loop that supports CSC maintenance and tumor growth. **(B)** Sympathetic nerve–driven immune exhaustion. Sympathetic nerve fibers release norepinephrine (NE) into the tumor microenvironment. NE engages β_2_-adrenergic receptors (β_2_-AR) on CD8^+^ T cells, increasing inhibitory checkpoint expression (e.g. PD-1), suppressing IFN-γ production and cytotoxic effector function, and thereby impairing antitumor immunity. In parallel, NE skews tumor-associated macrophages toward an M2-like, immunosuppressive phenotype, further promoting immune escape in colorectal cancer.

A complex “enteric neuron-serotonin-Wnt-CSC” self-reinforcing circuit exists in the colorectal cancer microenvironment, with the immune system playing a critical regulatory role in this circuit (18). We have summarized this effect in **Figure 3**.

Tumor-associated macrophages (TAMs) represent key participants in this circuit. 5-HT promotes M2-type polarization and immunosuppressive phenotypes through acting on 5-HT2B and 5-HT7 receptors on TAM surfaces (20). Anti-inflammatory factors such as IL-10 and TGF-β secreted by M2-type TAMs reinforce the immunosuppressive microenvironment, while secreted Wnt ligands such as Wnt3a and Wnt5a can activate Wnt signaling pathways in CSCs (51). Additionally, neurotrophic factors secreted by M2-type TAMs reciprocally promote enteric neuron survival and function, forming a positive feedback loop.

T cells in this circuit are also subject to multiple regulatory mechanisms. High 5-HT concentrations in the tumor environment primarily inhibit CD8^+^ T cell cytotoxic function through 5-HT2A receptors (20). Aberrant Wnt signaling activation can also directly influence T cell differentiation; high levels of β-catenin signaling promote Treg differentiation while inhibiting effector T cell function.

#### 5.3.2 Neurotransmitter regulation of immune cells

Neurotransmitters play critical regulatory roles in TAM polarization. Norepinephrine activates the cAMP/PKA signaling pathway through β2-AR, inhibiting M1-type marker expression such as iNOS and IL-12 while promoting M2-type marker expression such as Arg1 and CD206 (45). This polarization shift in the tumor microenvironment favors tumor growth and immune evasion.

Acetylcholine exerts anti-inflammatory effects through α7nAChR, a mechanism termed the “cholinergic anti-inflammatory pathway” (24). α7nAChR activation inhibits NF-κB signaling pathways, reducing production of pro-inflammatory factors IL-1β, TNF-α, and IL-6. In the colorectal cancer microenvironment, this mechanism may promote tumor progression by suppressing anti-tumor inflammatory responses.

#### 5.3.3 Norepinephrine and CD8^+^ T cell function

Norepinephrine (NE) inhibition of CD8^+^ T cell function plays important roles in colorectal cancer immune evasion. CD8^+^ T cells express β2-AR; NE activation of β2-AR leads to elevated intracellular cAMP levels, inhibiting T cell receptor signal transduction through PKA pathways (41).

Specifically, β2-AR signal activation inhibits ZAP70 and LAT phosphorylation, leading to reduced production of effector cytokines such as IL-2 and IFN-γ, with cytotoxic granule release being suppressed (41). Additionally, chronic β2-AR stimulation can upregulate inhibitory receptor expression such as PD-1 and TIM-3, promoting T cell exhaustion (19).

In murine colorectal cancer models, pharmacological β-AR signal blockade can significantly enhance CD8^+^ T cell anti-tumor activity and improve checkpoint inhibitor therapy efficacy (52). Clinical studies also demonstrate that colorectal cancer patients using β-receptor blockers exhibit significantly prolonged overall survival. These findings provide theoretical basis for combined application of β-receptor blockers and immunotherapy.

#### 5.3.4 Sensory nerves and tumor immunosurveillance

The role of sensory nerves in tumor immunosurveillance has received widespread attention in recent years. Research by Balood and colleagues suggests that nociceptive sensory neurons may influence tumor progression through regulating immune cell function (49).

Substance P represents an important neuropeptide released from sensory neurons, exerting pro-inflammatory effects through acting on NK1 receptors on neutrophil surfaces, enhancing neutrophil chemotaxis and reactive oxygen production (53). Calcitonin gene-related peptide (CGRP) primarily exerts anti-inflammatory effects, inhibiting neutrophil activation through cAMP-dependent mechanisms (14). In the tumor microenvironment, CGRP may promote tumor progression by suppressing neutrophil-mediated anti-tumor immune responses.

Regulatory T cells (Tregs) occupy a special position in neuro-immune interactions. Studies have found that sympathetic nervous signals can promote Treg recruitment to tumor tissue and functional activation (44). β2-AR activation upregulates Treg CXCR4 expression, enhancing their response to CXCL12 chemotactic gradients. Neuropeptides such as VIP and PACAP directly promote Treg survival and immunosuppressive function through VPAC receptors (40). These mechanisms collectively promote tumor immune evasion.

## 6 The neuro-immune axis in other gastrointestinal malignancies

### 6.1 Pancreatic cancer

#### 6.1.1 Molecular basis of high perineural invasion

Pancreatic cancer is among the solid tumors with the highest rates of perineural invasion (PNI), ranging from 80% to 100%, which significantly contributes to its aggressive behavior and dismal prognosis (54). Complex bidirectional interactions exist between pancreatic cancer cells and neural cells, forming a “neuro-tumor axis” (55).

Nerve growth factor (NGF) and its receptor TrkA are highly expressed in pancreatic cancer and represent key molecular determinants of PNI. Pancreatic cancer cells secrete abundant NGF, attracting TrkA-expressing nerve fibers, while NGF released by neural cells reciprocally promotes tumor cell migration toward nerve bundles (56). Additionally, other neurotrophic factor systems, including GDNF/RET and artemin/GFRα3, participate in PNI formation. The CXCL12/CXCR4 chemokine axis establishes a chemotactic gradient that guides tumor cell invasion along neural tracks (57).

#### 6.1.2 Differential regulation of the immune microenvironment by sympathetic and vagal nerves

The autonomic nervous system within the pancreatic cancer microenvironment profoundly influences immune cell function. Renz et al., in a study published in *Cancer Cell*, demonstrated that norepinephrine released by sympathetic nerves activates β-adrenergic receptor (β-AR) signaling to promote tumor cell proliferation, invasion, and metastasis, while concurrently suppressing the antitumor functions of CD8^+^ T cells and natural killer (NK) cells (58).

Mechanistic investigations have revealed that β-AR activation promotes M2-polarization of tumor-associated macrophages (TAMs) and accumulation of myeloid-derived suppressor cells (MDSCs), which suppress T cell function through secretion of IL-10, TGF-β, and arginase 1 (Arg1) (59). Sympathetic signaling also activates cancer-associated fibroblasts (CAFs), which create both physical and chemical barriers through dense desmoplastic stroma and secretion of immunosuppressive factors (60).

The role of vagal innervation in pancreatic cancer is more nuanced. In another study published in *Cancer Discovery*, Renz et al. found that vagal cholinergic signaling suppresses pancreatic cancer stem cell properties through M1 muscarinic receptors (61). The vagus nerve modulates the tumor immune microenvironment by regulating macrophage polarization and T cell function, with its net effect depending on disease stage and microenvironmental context.

#### 6.1.3 Synergistic mechanisms of neural remodeling and immune evasion

Extensive neural remodeling occurs within the pancreatic cancer microenvironment, including neurogenesis, increased nerve fiber density, and alterations in neural phenotype (62). This neural remodeling is intimately linked to immune evasion, forming synergistic pro-tumorigenic mechanisms.

Neurogenesis not only increases nerve fiber numbers but also alters neurotransmitter release patterns and the microenvironmental signaling landscape. Newly formed nerve fibers are predominantly sympathetic and sensory in nature, profoundly influencing immune cell distribution and function through release of norepinephrine, neuropeptide Y, and substance P (9). Saloman et al., in a study published in *PNAS*, demonstrated that sensory nerve ablation in a genetic mouse model of pancreatic cancer significantly delayed tumor initiation and progression (55).

Neural remodeling also indirectly influences the immune microenvironment by affecting angiogenesis and stromal remodeling. Studies have demonstrated that the neurovascular link within the tumor microenvironment represents an important mechanism of tumor progression, with adrenergic signaling capable of promoting angiogenesis through modulation of endothelial cell metabolism (63). In other solid tumors such as prostate cancer, adrenergic nerves have been shown to activate an angiogenic-metabolic switch. The hypoxic state of the pancreatic cancer microenvironment further promotes the recruitment and activation of immunosuppressive cells (64).

### 6.2 Hepatocellular carcinoma

#### 6.2.1 Hepatic autonomic innervation and immune regulation

The liver receives dual autonomic innervation, with sympathetic input primarily from the celiac ganglion and parasympathetic input predominantly from the vagus nerve (65). The liver possesses a unique immune microenvironment enriched with Kupffer cells (liver-resident macrophages), hepatic sinusoidal endothelial cells, and various lymphocyte subpopulations. Autonomic signaling regulates hepatic inflammation and tumorigenesis through modulation of these immune cell functions (66).

The liver is an immunologically tolerant organ, and sympathetic activation plays a crucial role in maintaining hepatic immune tolerance. In the context of hepatocellular carcinoma (HCC), this tolerance may be exploited by tumors to evade immune surveillance (67).

#### 6.2.2 Chronic stress and HCC progression

Chronic stress-induced sympathetic hyperactivation promotes HCC development and progression. In murine liver cancer models, chronic restraint stress significantly accelerates tumor growth, an effect reversible by β-adrenergic blockade (18). Mechanistic studies have shown that sympathetic signaling promotes M2 polarization of Kupffer cells via β2-AR, leading to secretion of immunosuppressive factors such as IL-10 and TGF-β, thereby suppressing CD8^+^ T cell antitumor function (45).

Sympathetic signaling also influences the hepatic microenvironment through regulation of hepatic stellate cells (HSCs). Studies in non-alcoholic fatty liver disease (NAFLD) have demonstrated that β-AR activation promotes HSC activation and fibrogenesis (68). This mechanism may play an important role during the progression from chronic liver disease to HCC, creating a pro-tumorigenic stromal microenvironment.

#### 6.2.3 Neuro-immune reprogramming in the context of chronic hepatitis

Chronic viral hepatitis (HBV and HCV infections) and non-alcoholic steatohepatitis (NASH) represent major risk factors for HCC (41). In the setting of these chronic liver diseases, hepatic innervation and the immune microenvironment undergo significant reprogramming.

Chronic inflammatory stimulation leads to increased hepatic sympathetic nerve density and enhanced neurotransmitter release. Concurrently, hepatic immune cells exhibit altered expression of neurotransmitter receptors, increasing their sensitivity to neural signals [18]. During the progression from NASH to HCC, lipotoxicity and inflammation-induced sympathetic activation promotes hepatic inflammatory responses while simultaneously inhibiting lipid metabolism, creating a vicious cycle (69).

### 6.3 Esophageal cancer

#### 6.3.1 Vagal innervation and the esophageal cancer microenvironment

The esophagus receives abundant vagal innervation. In multiple gastrointestinal malignancies, acetylcholine has been demonstrated to promote tumor cell proliferation and invasion through activation of M3 muscarinic receptors. Yang et al. confirmed in gastric cancer research that acetylcholine activates the EGFR signaling pathway through M3 receptors to promote tumor cell proliferation (70). Similar mechanisms may exist in esophageal cancer. Additionally, cholinergic signaling modulates immune cell function within the tumor microenvironment via α7 nicotinic acetylcholine receptors (α7nAChR), a mechanism that has been demonstrated in multiple tumor types to promote M2 TAM polarization and establishment of an immunosuppressive microenvironment (16).

#### 6.3.2 Neuroendocrine differentiation and immune phenotype

Esophageal neuroendocrine carcinoma (NEC) represents a rare but highly aggressive tumor subtype expressing neuroendocrine markers including synaptophysin, chromogranin A, and CD56 (71). Esophageal NEC exhibits distinctive immune microenvironment characteristics with an overall immunosuppressive phenotype. Tumor cell-secreted neuropeptides such as somatostatin and vasoactive intestinal peptide (VIP) directly regulate immune cell function, promoting immune evasion (72).

### 6.4 Commonalities and differences across tumor types

#### 6.4.1 Heterogeneity of the neuro-immune axis across GI malignancies

Although neuro-immune interactions represent a common feature of GI malignancies, significant heterogeneity exists among different tumor types (73).

Regarding neural innervation, pancreatic cancer exhibits the highest PNI rate and greatest nerve fiber density; colorectal cancer shows more prominent enteric nervous system (ENS) involvement; and vagal innervation plays a more significant role in esophageal and gastric cancers (33). With respect to the immune microenvironment, colorectal cancer displays the most prominent inflammatory features; pancreatic cancer exhibits the most severe stromal components and immunosuppression; and HCC possesses unique immune tolerance characteristics (13).

Concerning neurotransmitter composition, serotonin (5-HT) plays the most important role in colorectal cancer, NGF/neurotrophin signaling is prominent in gastric cancer, and sympathetic signaling exerts more pronounced pro-tumorigenic effects in pancreatic cancer and HCC (74). These differences suggest that distinct neuro-immune targeting strategies may be required for different GI malignancy types.

#### 6.4.2 Influence of anatomical and histological features

The anatomical and histological characteristics of different GI tract segments profoundly influence neuro-immune interaction patterns (75). The colorectum possesses a complex ENS network, enabling more direct and intimate tripartite communication among enteric neurons, tumor cells, and immune cells. In contrast, although the pancreas lacks an ENS, its abundant peripheral innervation and highly dense stroma provide a unique spatial architecture for neuro-immune interactions (60).

The gut microbiota represents an important factor influencing the immune microenvironment of GI malignancies, and bidirectional regulatory relationships exist between the nervous system and the microbiota (76). In colorectal cancer, the gut microbiota modulates ENS function and immune responses through production of short-chain fatty acids and secondary bile acids. During the progression from chronic liver disease to HCC, the gut-liver axis and microbial dysbiosis also play important roles (77).

Lymphatic drainage patterns and local immune tissue architecture also influence neuro-immune interactions. The intestine is enriched with gut-associated lymphoid tissue (GALT), and these local immune structures maintain intimate spatial relationships and functional connections with the ENS (75). Understanding these anatomical and histological features will facilitate the design of more precise therapeutic strategies that exploit or block site-specific neuro-immune interactions.

## 7 Therapeutic innovations and clinical translation

### 7.1 Advances in immunotherapy

#### 7.1.1 Immune checkpoint inhibitors

Immune checkpoint inhibitors (ICIs) have revolutionized the treatment landscape of gastrointestinal malignancies. In colorectal cancer, the KEYNOTE-177 trial demonstrated that first-line pembrolizumab significantly improved median progression-free survival (PFS) compared with chemotherapy (16.5 months vs. 8.2 months) in patients with microsatellite instability-high (MSI-H) or mismatch repair-deficient (dMMR) metastatic colorectal cancer (54). However, MSI-H/dMMR accounts for only approximately 5% of metastatic colorectal cancer cases, and single-agent immunotherapy demonstrates limited efficacy in the majority of patients with microsatellite-stable (MSS) disease (55).

Significant advances have also been achieved in immunotherapy for gastric and gastroesophageal junction cancers. The CheckMate-649 trial confirmed that first-line nivolumab combined with chemotherapy significantly improved overall survival (OS) in patients with HER2-negative advanced gastric cancer with PD-L1 combined positive score (CPS) ≥5 (56). In esophageal cancer, the CheckMate-577 trial established a new paradigm of adjuvant immunotherapy following neoadjuvant chemoradiotherapy (57).

Immunotherapy for hepatocellular carcinoma (HCC) has achieved landmark breakthroughs. The IMbrave150 trial demonstrated that atezolizumab combined with bevacizumab significantly improved both OS and PFS compared with sorafenib as first-line treatment for unresectable HCC, representing the first regimen to surpass sorafenib (58). This “immunotherapy plus anti-angiogenesis” combination strategy provides a valuable paradigm for other GI malignancies.

Pancreatic cancer remains the most challenging tumor type for immunotherapy. Due to its highly immunosuppressive microenvironment and dense desmoplastic stroma, single-agent immunotherapy has demonstrated extremely limited efficacy (59). Current research focuses on exploring combination treatment strategies. Notably, nervous system-related biomarkers such as nerve fiber density and neurotransmitter receptor expression may provide new dimensions for patient stratification (60).

#### 7.1.2 Cellular immunotherapy

CAR-T cell therapy faces multiple challenges in solid tumors, particularly GI malignancies, including the lack of tumor-specific target antigens, immunosuppressive microenvironment, and physical barriers inherent to solid tumors (61). CAR-T research targeting GI tumors has explored multiple targets: Claudin 18.2 is highly expressed in gastric cancer with restricted expression in normal tissues, representing a promising target. Qi et al. reported encouraging efficacy in a phase I trial published in *Nature Medicine (*62).

To overcome the barriers posed by the solid tumor microenvironment, next-generation CAR-T technologies have emerged. Armored CAR-T cells are genetically engineered to secrete immunostimulatory factors or express chemokine receptors to enhance tumor homing (9). Bispecific T-cell engagers (BiTEs) represent another innovative strategy that can recruit T cells to the vicinity of tumor cells and activate their cytotoxic function (63).

#### 7.1.3 Macrophage-targeted therapy

Tumor-associated macrophages (TAMs) play critical pro-tumorigenic roles in the GI tumor microenvironment, making TAM targeting an emerging therapeutic strategy (64). CSF1R inhibitors are among the most extensively studied TAM-targeting agents, with multiple CSF1R inhibitors currently in clinical trials (65). In pancreatic cancer models, CSF1/CSF1R blockade reprograms tumor-infiltrating macrophages and improves response to T-cell checkpoint immunotherapy (66).

M2-to-M1 repolarization strategies aim to reverse the immunosuppressive phenotype of TAMs. PI3Kγ inhibitors promote M1 polarization by blocking PI3Kγ signaling and have demonstrated synergistic effects when combined with immune checkpoint inhibitors in pancreatic cancer models (67). Notably, neurotransmitters profoundly influence macrophage polarization: acetylcholine via α7nAChR and norepinephrine via β2-AR both promote M2 polarization (18). Therefore, combining neurotransmitter pathway inhibitors with macrophage-targeted therapies may produce synergistic effects.

### 7.2 Neural-targeted therapy

#### 7.2.1 β-Adrenergic receptor blockers

**Figure 4:**
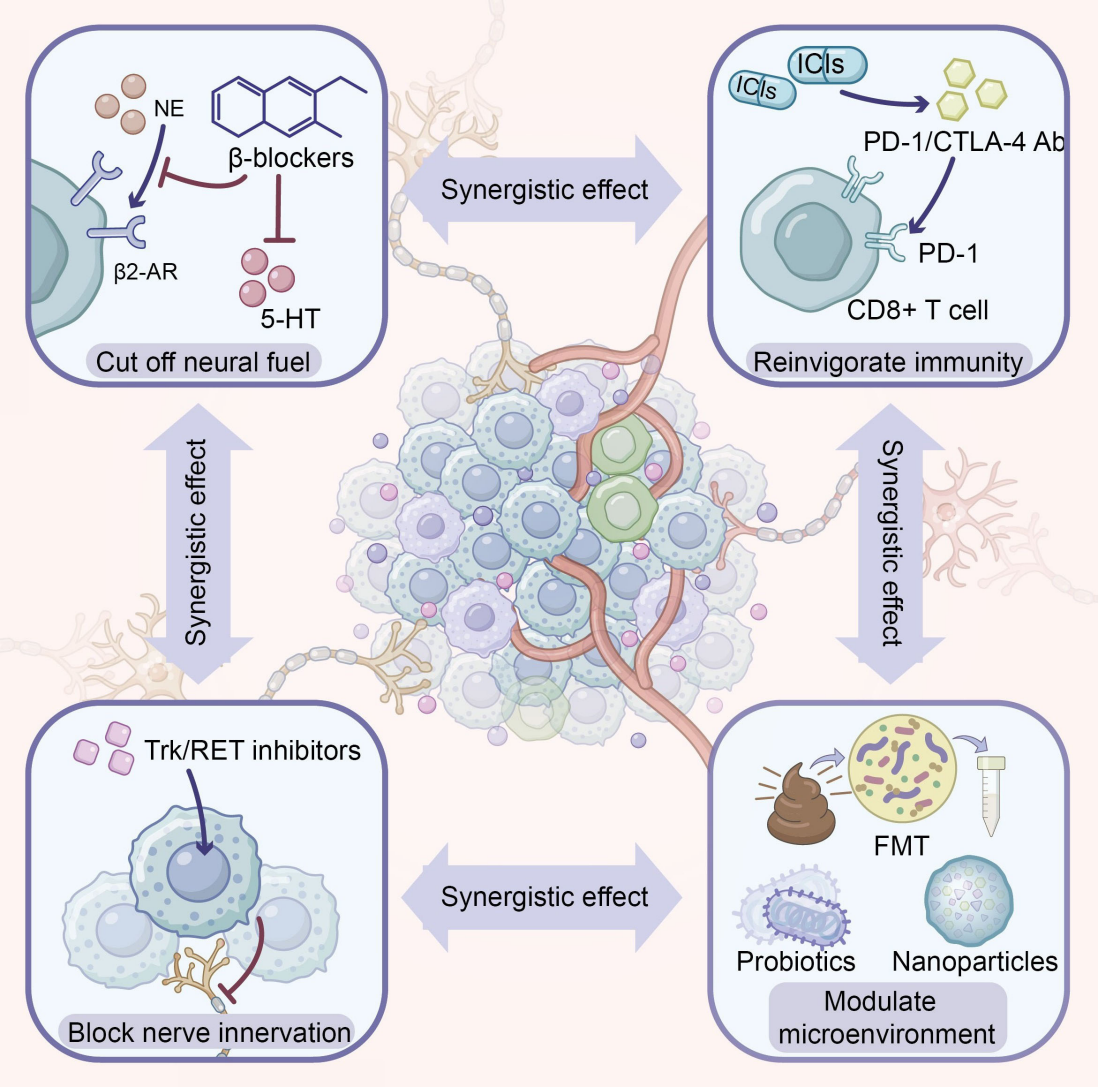
Neuro-targeted combination strategies in gastrointestinal cancer. Central illustration depicts a neuro-innervated tumor microenvironment composed of malignant cells, infiltrating immune cells, nerve fibers and blood vessels. Surrounding panels summarize candidate therapeutic interventions that target distinct components of this network and may act synergistically. Upper left: β-adrenergic receptor blockers and 5-HT receptor antagonists interrupt sympathetic neurotransmission by blocking β_2_-AR–mediated norepinephrine (NE) signaling and serotonin (5-HT) inputs, thereby “cutting off neural fuel” to the tumor. Upper right: Immune checkpoint inhibitors (ICIs), including anti-PD-1 and anti-CTLA-4 antibodies, bind inhibitory receptors on CD8^+^ T cells, restore effector cytokine production and cytotoxicity, and “reinvigorate immunity”. Lower left: Trk and RET inhibitors directly target neurotrophic signaling pathways that drive tumor–nerve crosstalk, thereby “blocking nerve innervation” and tumor growth support. Lower right: Microenvironment-modulating approaches such as fecal microbiota transplantation (FMT), probiotics and nanoparticle-based delivery systems reshape the gut microbiota and local milieu to “modulate the microenvironment” and enhance host anti-tumor responses. Bidirectional arrows between panels highlight the potential synergistic effects of combining neural blockade, direct neurotrophic inhibition, immune checkpoint blockade and microbiome-targeted interventions.

In **Figure 4**, we provide a detailed discussion on potential synergistic therapeutic approaches targeting neural pathways. The potential of β-adrenergic receptor blockers (β-blockers) in cancer treatment has garnered increasing attention. Numerous retrospective studies and meta-analyses have demonstrated improved prognosis among β-blocker users across multiple tumor types (45). A meta-analysis including over 10,000 colorectal cancer patients showed that β-blocker use was significantly associated with prolonged overall survival (HR = 0.84, 95% CI: 0.77-0.92) (68).

The antitumor mechanisms of β-blockers are multifaceted. Beyond direct inhibition of tumor cell proliferation and metastasis, β-blockers exert effects through modulation of the immune microenvironment: reversing M2 polarization of TAMs, enhancing antitumor functions of CD8^+^ T cells and NK cells, and reducing the immunosuppressive effects of MDSCs (41). Additionally, studies have demonstrated that β-blockers can inhibit tumor angiogenesis through downregulation of VEGF expression (69).

Prospective clinical trials are exploring the application of β-blockers in cancer treatment. A phase II clinical trial evaluating propranolol combined with chemotherapy in advanced pancreatic cancer showed a trend toward prolonged median survival in the combination treatment group (70).

#### 7.2.2 5-HT receptor antagonists

The 5-HT/HTR2B signaling pathway plays an important role in colorectal cancer stem cell maintenance (16). Lee et al., in a study published in *Biomedicine & Pharmacotherapy*, demonstrated that the selective HTR2B antagonist SB204741 and the novel inhibitor GM-60186 significantly inhibited colorectal cancer cell proliferation and migration in both *in vitro* and xenograft mouse models, with the mechanism involving suppression of ERK signaling (71). Several marketed 5-HT receptor antagonists have been associated with improved tumor prognosis in retrospective studies, providing rationale for drug repurposing.

#### 7.2.3 Neurotrophin inhibition

The NGF/TrkA pathway represents a critical axis in nervous system-tumor interactions. TrkA inhibitors larotrectinib and entrectinib have received FDA approval for the treatment of NTRK gene fusion-positive solid tumors (72). Although NTRK fusions are relatively rare in GI tumors (approximately 0.5-1% in colorectal cancer), TRK inhibitors demonstrate impressive efficacy in these patients, with overall response rates exceeding 75% (73).

Beyond patients harboring NTRK fusions, blocking the NGF/TrkA signaling pathway may benefit a broader population of GI tumor patients. Hayakawa et al., in a study published in *Cancer Cell*, demonstrated that NGF promotes gastric tumorigenesis through aberrant cholinergic signaling (33). In pancreatic and gastric cancers with significant perineural invasion, NGF blockade may suppress tumor progression by disrupting nerve-tumor interactions (13).

The GDNF/RET signaling pathway also plays an important role in perineural invasion. RET inhibitors such as selpercatinib and pralsetinib have been approved for RET fusion-positive or mutant tumors (74). In pancreatic cancer, GDNF expression is associated with perineural invasion and poor prognosis.

#### 7.2.4 Denervation strategies

Chemical denervation using 6-OHDA or capsaicin selectively destroys specific types of nerve fibers. Saloman et al., in a study published in *PNAS*, demonstrated that sensory nerve ablation in a genetic mouse model of pancreatic cancer significantly delayed tumor initiation and progression (75). However, the clinical application of chemical denervation is limited by safety and specificity concerns.

The role of the vagus nerve may differ across disease stages and tumor types. On one hand, vagotomy may reduce the risk of certain tumor development; on the other hand, the cholinergic anti-inflammatory pathway of the vagus nerve may play a protective role in controlling chronic inflammation and preventing carcinogenesis (76). Future studies with greater precision are needed to guide individualized treatment decisions.

### 7.3 Combination strategies: targeting the neuro-immune axis

#### 7.3.1 Theoretical rationale and synergistic potential

The combination of immune checkpoint inhibitors with neuromodulatory agents represents an innovative strategy for targeting the neuro-immune axis (77). The theoretical rationale is based on the observation that the nervous system suppresses antitumor immune responses through multiple mechanisms, including promoting the recruitment and activation of immunosuppressive cells, inhibiting the function of effector T cells and NK cells, and regulating the expression of immune checkpoint molecules (10).

Norepinephrine released by sympathetic nerves suppresses the cytotoxic function of CD8^+^ T cells through β-AR signaling while simultaneously upregulating expression of inhibitory receptors such as PD-1 (22). Therefore, β-blockers may enhance the efficacy of immune checkpoint inhibitors by relieving nervous system-mediated suppression of T cells. Bucsek et al., in a study published in *Cancer Research*, demonstrated that β-blockers combined with anti-PD-1 antibodies significantly improved tumor response rates and survival in a murine colorectal cancer model (52).

Inhibition of the serotonin/HTR2B pathway may also enhance immunotherapy efficacy. HTR2B antagonists not only directly inhibit tumor stem cells but may also improve the tumor immune microenvironment by reducing recruitment of immunosuppressive cells (19).

#### 7.3.2 Preliminary clinical evidence and personalized protocol design

Although combination therapies targeting the neuro-immune axis remain in early stages, preliminary clinical evidence is encouraging. Gandhi et al., in a phase I clinical trial published in *Clinical Cancer Research*, confirmed that propranolol combined with pembrolizumab was safe and tolerable in patients with locally advanced and metastatic melanoma, with an observed objective response rate of 78% (78). In the GI tumor field, a phase II clinical trial is evaluating the efficacy of propranolol combined with pembrolizumab in metastatic colorectal cancer (NCT03245854).

The design of personalized combination treatment protocols requires consideration of multiple factors (7). First, tumor type and stage are key determinants: the pro-tumorigenic role of sympathetic nerves is more prominent in pancreatic cancer, making β-blockers a potentially preferred option, whereas in colorectal cancer, inhibition of the serotonin pathway may be more important. Second, biomarker-guided patient stratification is crucial, requiring detection of nerve fiber density, neurotransmitter receptor expression, and immune cell infiltration characteristics in tumor tissue to identify patients most likely to benefit. Third, treatment timing and sequencing require optimization; preclinical studies suggest that neuromodulation can reshape the immune status of the tumor microenvironment, but optimal treatment sequencing strategies require further exploration (79).

### 7.4 Emerging therapeutic modalities

#### 7.4.1 Gut microbiome modulation

The gut microbiota plays a critical bridging role in the “gut-brain axis” and “gut-immune axis” (80). Studies have shown that gut microbiome composition influences the efficacy of immune checkpoint inhibitors. Routy et al., in a study published in *Science*, demonstrated that enrichment of certain “beneficial” bacterial genera (such as *Akkermansia muciniphila*) was associated with better immunotherapy responses (81).

Fecal microbiota transplantation (FMT) has been employed in some studies to enhance immunotherapy efficacy. Baruch et al. and Davar et al. independently published studies in *Science* showing that some melanoma patients who were previously unresponsive to immunotherapy achieved clinical benefit after receiving FMT from responders (82, 83). This strategy provides a reference for similar research in GI tumors.

Dietary intervention represents another accessible modulatory approach. Fiber-rich diets may improve intestinal immune function and neural regulation through increased production of short-chain fatty acids (84).

#### 7.4.2 Nanotechnology and neuromodulation devices

Nanomedicine delivery systems provide a technological platform for precise targeting of neuro-immune interactions within the tumor microenvironment (85). TAM-targeting nanosystems can selectively deliver immunomodulatory agents to TAMs, achieving macrophage reprogramming. Multifunctional nanoplatforms can simultaneously deliver immune checkpoint inhibitors and β-blockers, maximizing synergistic effects.

Vagus nerve stimulation (VNS) devices have attracted attention in the oncology field due to their anti-inflammatory and immunomodulatory effects (86). Theoretically, appropriate VNS may exert antitumor effects through mechanisms including reduced chronic inflammation, improved metabolic function, and enhanced immune surveillance. However, excessive cholinergic signaling may promote immunosuppression within the tumor microenvironment, making optimization of VNS parameters crucial (87).

### 7.5 Clinical trial progress and future directions

Multiple clinical trials are currently evaluating therapeutic strategies targeting the neuro-immune axis. Trials combining immune checkpoint inhibitors with β-blockers include: NCT03245854, evaluating propranolol combined with pembrolizumab in MSS colorectal cancer; and NCT04596241, evaluating the safety and efficacy of carvedilol combined with nivolumab in pancreatic cancer.

Translation from basic research to clinical application still faces multiple challenges (77). Target validation and mechanism elucidation represent primary challenges, requiring validation of key targets and pathways in human tissues and clinical samples. Application of cutting-edge technologies such as single-cell multi-omics and spatial transcriptomics will accelerate this process (88).

Development and validation of biomarkers are key to achieving precision medicine. Liquid biopsy technologies may provide tools for non-invasive monitoring. Drug development and optimization require interdisciplinary collaboration; many neuromodulatory drugs were originally developed for other indications, and their dosing and administration schedules in oncology require re-optimization (89).

Clinical trial design requires innovation. Novel research approaches such as adaptive trial designs, basket/umbrella trials, and real-world studies may be more appropriate. Furthermore, since neuro-immune interventions may exert effects through symptom improvement and quality of life enhancement, endpoint selection should be comprehensive, including not only traditional oncology endpoints (OS, PFS) but also patient-reported outcome measures (90).

## 8 Discussion

In recent years, the neuro-immune axis in gastrointestinal tumors has emerged as a promising area of research. The nervous and immune systems engage in complex bidirectional interactions within the tumor microenvironment, providing a theoretical foundation for developing novel therapeutic strategies (10). However, existing evidence has notable limitations. Current research predominantly derives from animal models, and differences exist between mice and humans in enteric nervous system architecture. Single-cell resolution studies have revealed both similarities and differences in neuronal subtype composition between species, which may impact the translatability of research findings (91). At the clinical evidence level, although multiple meta-analyses have demonstrated associations between β-blocker use and improved prognosis in various cancers, these observational studies are susceptible to confounding factors such as immortal time bias, and high-quality prospective randomized controlled trials remain lacking (68).

Different types of GI malignancies exhibit significant heterogeneity in neuro-immune interactions. Pancreatic cancer, with its extremely high rate of perineural invasion (80-100%) and dense desmoplastic stroma, represents the disease type with the greatest intervention potential, with neural plasticity playing a critical role in both pancreatitis and pancreatic cancer (42). Therapeutic opportunities in colorectal cancer primarily center on modulation of the enteric nervous system-serotonin axis, with the 5-HT signaling pathway playing a key role in cancer stem cell maintenance (16). Therapeutic strategies for gastric cancer may focus on neurotrophin signaling pathways and the CGRP-RAMP1 axis, through which nociceptive neurons promote gastric tumor progression (14). Hepatocellular carcinoma requires consideration of the liver’s unique characteristics as an immunologically tolerant organ, with sympathetic activation playing an important role in maintaining hepatic immune tolerance (92).

The inherent limitations of animal models represent a primary concern. Differences exist between species in the distribution of intestinal neuronal subtypes and neurotransmitter receptor expression patterns, which have been confirmed through single-cell sequencing studies (91). The complexity and dynamic evolution of the tumor microenvironment pose additional challenges for clinical translation, with the microenvironment continuously remodeling from tumor initiation through metastatic dissemination (7). Clinical translation faces multiple obstacles. The lack of biomarkers represents the greatest challenge; currently proposed potential markers such as tumor nerve fiber density and neurotransmitter receptor expression levels mostly lack standardized detection methods and prospective validation (77). Patient stratification difficulties stem from tumor heterogeneity; even within the same tumor type, different patients may exhibit significant variation in innervation patterns and immune microenvironment characteristics (18).

Notably, the literature presents complex evidence regarding nervous system effects. The traditional view holds that sympathetic nerves regulate the tumor microenvironment through β-adrenergic signaling, promoting angiogenesis, immunosuppression, and tumor progression (45). However, recent studies have revealed the complexity of neural regulation. Balood et al., in a study published in *Nature*, demonstrated that nociceptor neurons (a class of sensory neurons) exert important effects on tumor immunosurveillance, with their ablation promoting tumor growth and metastasis (49). These findings suggest that nervous system regulation of tumors is highly complex, with different neuronal types potentially exerting different or even opposing effects, and the net outcome depending on tumor type, disease stage, and microenvironmental context.

The combination of single-cell sequencing with spatial transcriptomics technologies provides unprecedented resolution for understanding the tripartite dialogue among nerves, immune cells, and tumors (93). In pancreatic cancer research, the integration of spatial transcriptomics with single-cell RNA sequencing has successfully revealed the complexity of tumor tissue architecture (88). Through ligand-receptor interaction analysis, critical neuro-immune interaction axes can be identified. The development of organ-on-chip and organoid models provides new platforms for mechanistic studies and drug screening. Patient-derived organoids can recapitulate the genetic diversity and microenvironmental complexity of tumors *in vitro*, providing tools for developing individualized treatment strategies (94). Artificial intelligence-assisted multi-omics integration analysis can identify patterns, discover biomarkers, and predict treatment responses from complex data.

Future clinical research should focus on establishing multicenter prospective cohorts to systematically collect patient clinical information and biological samples for standardized testing of neuro-immune-related biomarkers. Regarding RCT design, drugs with existing clinical experience such as β-blockers and 5-HT receptor antagonists can relatively rapidly enter clinical trials for oncology indications. Implementing neuro-immune targeted therapy in clinical practice requires establishing an individualized decision-making framework: first, identifying patients likely to benefit through comprehensive baseline assessment; second, considering patient comorbidities and medication history; third, adopting a stepwise treatment strategy. Preclinical studies have demonstrated that β-blockers can reduce tumor growth and enhance response to anti-CTLA4 therapy by modulating the tumor microenvironment (69), providing theoretical support for combining neuromodulatory agents with immune checkpoint inhibitors; and fourth, establishing multidisciplinary team collaboration models.

## 9 Conclusion

This review systematically examines the role of the neuro-immune axis in GI malignancies. Core findings include: extensive bidirectional interactions exist between the nervous and immune systems within the tumor microenvironment; different types of GI tumors exhibit distinct neuro-immune interaction patterns, such as the high degree of perineural invasion in pancreatic cancer, the enteric nervous system-serotonin-Wnt axis in colorectal cancer, and the CGRP-RAMP1 axis-driven mechanisms in gastric cancer (10, 14, 16); therapeutic strategies targeting the neuro-immune axis demonstrate tremendous potential, including combination of immune checkpoint inhibitors with neuromodulatory agents, synergy between macrophage-targeted therapy and neural signal blockade, and emerging nano-delivery systems (157, 95).

The translational potential of neuro-immune axis-targeted therapy is manifested at multiple levels. From a biological perspective, targeting this axis not only directly inhibits tumor growth but may also enhance the efficacy of other treatments by improving the immune microenvironment. From a clinical practice perspective, many neuromodulatory drugs have extensive clinical experience, and drug repurposing strategies can significantly shorten development timelines. From a precision medicine perspective, neuro-immune-related biomarkers can help identify patient subgroups most likely to benefit.

Looking ahead, this field will develop toward greater refinement, systematization, and translation. At the basic research level, cutting-edge technologies such as single-cell multi-omics and spatial transcriptomics should be utilized to elucidate molecular mechanisms in depth; at the translational research level, human tissue sample studies should be strengthened to validate animal model findings; at the clinical research level, high-quality prospective trials should be designed to evaluate the efficacy and safety of combination treatments. Through the close integration of basic, translational, and clinical research, neuro-immune axis-targeted therapy is poised to become an important component of comprehensive GI tumor treatment, bringing better therapeutic outcomes and quality of life to patients.
